# The Incidence and Severity of COVID-19 in the Liverpool Severe Asthma Population Undergoing Biologic Therapy

**DOI:** 10.7759/cureus.28790

**Published:** 2022-09-05

**Authors:** Yahya Abdullah

**Affiliations:** 1 Department of Respiratory Medicine, Liverpool University Hospitals NHS Foundation Trust, Liverpool, GBR

**Keywords:** liverpool, medical icu, covid-19 severe infection, biologic therapies, monoclonal antibody, severe asthma, covid-19

## Abstract

Background

The coronavirus disease 2019 (COVID-19) infection can have a variable impact on patients. Various host factors have been identified that play a significant role in the risk of COVID-19 infection and its severity. Patients with severe asthma have been clinically vulnerable since the first wave of the pandemic and the resurgence of COVID-19 in the United Kingdom in January 2020. In addition, those on treatment with monoclonal antibodies (mAbs) have been identified as being vulnerable to COVID-19 infection and severity by the World Health Organization and the Department of Health. However, the evidence to support this notion is limited, and there has been contrary evidence to suggest severe asthma is protective against COVID-19. In this study, a retrospective review of severe asthma patients in the Liverpool population between 1st January 2020 and 31st January 2021 was conducted. This study aimed to determine the association between asthma severity and the risk of COVID-19 infection and/or its severity for patients on mAb treatment.

Methodology

We conducted a review of all patients from the Liverpool severe asthma database/spreadsheet who tested positive in the community and at the hospital. Admission records, primary records, emails, and microbiological data for Anglia ICE were reviewed at the Royal Liverpool and Aintree University Hospital. A COVID-19 diagnosis was predefined as a positive lateral flow test and a positive polymerase chain reaction test. The proportion of patients with COVID-19 pneumonia and severe COVID-19 disease requiring hospital admission and escalation to intensive care (observation, intubation, continuous positive airway pressure) was noted. Other patient characteristics were recorded including age, weight, body mass index (BMI), gender, smoking status (never, former, current smoker), bronchiectasis, and the forced expiratory volume.

Results

In total, 760 patients were identified to have severe asthma, of whom 59 (7.8%) tested positive for COVID-19 and 701 (92.2%) tested negative. A total of 244 (32%) patients were taking mAbs, and 516 (68%) were not on mAb treatment. Patients were more susceptible to COVID-19 on an mAb (13.5%) versus non-mAb (5%) (odds ratio (OR) = 2.95; 95% confidence interval (CI) = 1.72 to 5.05) . A larger proportion of severe asthma patients on mAb treatment testing positive for COVID-19 were current smokers and had a higher BMI. Furthermore, severe asthmatics taking mAbs did not have a higher risk of severe COVID-19 disease, hospitalisation, and intensive care admission.

Conclusions

In the Liverpool severe asthma population, patients undergoing mAb therapy had a higher incidence of COVID-19 compared to non-mAb groups; however, they were not at a higher risk of severe disease progression. These findings suggest that continuing biologic therapy in severe asthmatics with COVID-19 appears to be safe to prevent exacerbations.

## Introduction

There were 10 million cases of coronavirus disease 2019 (COVID-19) and 500,000 deaths within the first six months of the first wave of the pandemic [1]. Patients presenting with comorbidities are known to have a higher risk of progressing to severe COVID-19 and hospitalisation [2]. A meta-analysis review showed that 50% of patients diagnosed with Middle Eastern respiratory syndrome (MERS) presented with pre-existing diabetes and hypertension, 30% presented with cardiac disease, and 16% were classed as obese [3]. These findings are consistent with current evidence which demonstrates the above conditions promote the downregulation of proinflammatory cytokines and dysregulate the innate and adaptive immune system making the host susceptible to infection with a poor prognosis [4,5]. While the aforementioned conditions have been linked to hospitalisation and severe COVID-19, few studies are available concerning the vulnerability of asthma patients and COVID-19 infection and severity. The available literature available is conflicting. In addition, studies concerning the role of treatment with monoclonal antibodies (mAbs) in severe asthma patients and the risk of severe COVID-19 are also limited [6]. This justifies and warrants further investigation.

Different mAbs target specific inflammatory pathways in eosinophilic inflammatory type 2 asthma and exert different effects. Mepolizumab and reslizumab exert their effects by downregulating the differentiation and activation of eosinophils through interaction of the interleukin (IL)-5 cytokines. Benralizumab acts as an IL-5 receptor antagonist and can activate natural killer cells, thus, promoting eosinophil apoptosis. Omalizumab prevents the release of mediators that are responsible for bronchospasm by blocking the interaction of immunoglobulin E and receptors located on mast and basophil cells. Dupilumab inhibits the release of proinflammatory cytokines by disrupting the IL-4 and IL-13 signalling pathways.

The current understanding is respiratory viruses such as coronaviruses are implicated in the exacerbation of asthma partly due to the association of bronchial inflammation [7-9]. However, there are conflicting studies that suggest asthma can have a protective role against COVID-19 through a dominant Th2 environment [10]. To unravel the clinical heterogeneity in this patient cohort, the risk of COVID-19 infection and severity in the severe asthma population was examined with respect to mAb treatment.

## Materials and methods

Retrospective data were collected from the Royal Liverpool and Aintree University Hospital between 1st January 2020 and 31st January 2021. The inclusion criterion was set to include all severe asthma patients attending the Liverpool asthma service. Patients had a confirmed diagnosis of severe asthma according to the British Thoracic Society. Patients residing outside of the Liverpool area, nonadherence to treatment, and patients testing positive for COVID-19 outside the set date were excluded. The data were collected from the dashboard, and admission records were reviewed from the Royal Liverpool and Aintree University Hospitals. Finally, the data were collated onto a spreadsheet using Microsoft Excel. To further this end, primary records were reviewed using eExchange to confirm patients testing positive or negative in the community for COVID-19. Correspondence emails were reviewed and sent by specialist asthma nurses, general practitioners, and patients that informed if they had tested positive for COVID-19 or were admitted elsewhere. To allow for a robust mode of data collection, microbiological data for Anglia ICE was utilised for confirmation of the positivity or negativity of COVID-19 swab tests. A COVID-19 diagnosis was predefined as a positive lateral flow test and a positive polymerase chain reaction (PCR) test for severe acute respiratory syndrome coronavirus 2 (SARS-CoV-2). Other patient characteristics were recorded that included age, weight, body mass index (BMI), gender, smoking status (never, former, current smoker), bronchiectasis, and forced expiratory volume (FEV1). The study was approved by the local ethics committee of the Royal Liverpool and Aintree University Hospital, and all patients provided their consent to be part of the study.

The incidence of COVID-19 was recorded in the severe asthma population. The number of patients testing positive in the community or hospital was noted as well as the confirmation of a positive swab. The proportion of patients who had COVID-19 pneumonia and severe COVID-19 was also recorded. To further this end, patients requiring either admission for COVID-19 or escalation to the intensive care unit (ICU) for observation, intubation, and continuous positive airway pressure (CPAP) were recorded. In addition, the number of patients requiring CPAP on the ward was documented. Following this, the number of severe asthma patients currently on mAbs and not on mAbs was calculated. The proportion of severe asthma patients on mAbs who tested positive or negative for COVID-19 was subsequently calculated. This was compared to the number of severe asthma patients not on mAbs who tested positive or negative for COVID-19. The average age, average weight, average BMI, gender, bronchiectasis, smoking status, and average FEV1 were calculated in patients testing positive or negative for COVID-19 with respect to the severe asthma population, severe asthma population on mAbs, and severe asthma population not on mAbs. An odds ratio (OR) was calculated with a 95% confidence interval (CI) taken as statistically significant. Because a small number of COVID-19 cases were anticipated in the Liverpool severe asthma population, regression analysis for adjustment of confounders was not planned.

## Results

Incidence of COVID-19 and disease course

Between 1st January 2020 and 31st January 2021, there were a total of 760 patients in the Liverpool severe asthma database. Of the 760 severe asthma patients, 59 (7.8%) tested positive and were laboratory confirmed for COVID-19 infection, and 701 (92.2%) tested negative. Overall, 27 of the 59 (45.8%) patients tested positive for COVID-19 in the hospital compared to 32 (54.2%) in the community. In total, 13 of the 59 (22%) patients who tested positive for COVID-19 developed COVID-19 pneumonia; 14 of the 59 (23.7%) patients progressed to severe COVID-19 disease; 22 of the 59 (37.3%) patients required admission; six (10.2%) patients received CPAP on the ward; and two (3.4%) patients of the 59 who tested positive for COVID-19 required ICU admission, both of whom received CPAP. This is summarised in Table 1.

**Table 1:** The incidence of COVID-19 and disease progression in the Liverpool severe asthma population. COVID-19: coronavirus disease 2019; CPAP: continuous positive airway pressure; ICU: intensive care unit

Liverpool severe asthma population	Number of patients testing positive for COVID-19	Number of patients testing negative for COVID-19	Number of patients testing positive in the hospital	Number of patients testing positive in the community	Number of patients with COVID-19 pneumonia	Number of patients with severe COVID-19	Number of patients requiring admission	Number of patients receiving CPAP in the ward	Number of patients requiring ICU admission
760	59	701	27	32	13	14	22	6	2

The effect of mAb treatment on the incidence and severity of COVID-19

Of the 760 patients identified from the Liverpool severe asthma database, 244 (32%) were on mAbs and 516 (68%) were not on mAbs. In total, 88 (36%) patients were on benralizumab, four (2%) were on dupilumab, 86 (35%) were on mepolizumab, 63 (26%) were on omalizumab, and three (1%) were on reslizumab. Of the 244 severe asthma patients on mAbs, 33 (13.5%) tested positive and confirmed for COVID-19 whereas 211 (86.5%) tested negative for COVID-19. In addition, of the 516 severe asthma patients not taking mAbs, 26 (5%) tested positive for COVID-19 compared to 490 (95%) who tested negative. This is summarised in Table 2.

**Table 2:** The incidence of COVID-19 and the disease progression for the Liverpool severe asthma population undergoing mAb therapy. COVID-19: coronavirus disease 2019; mAb: monoclonal antibody

	COVID-19 infection	Severe asthma patients on mAb	Severe asthma patients on mAb positive for COVID-19	Severe asthma patients not on mAb positive for COVID-19	Severe asthma patients on mAb positive for COVID-19 progressing to severe COVID-19	Severe asthma patients not on mAb positive for COVID-19 progressing to severe COVID-19	Severe asthma patients on mAb positive for COVID-19 needing admission	Severe asthma patients not on mAb positive for COVID-19 needing admission
Yes	59	244	33	26	4	10	8	14
No	701	516	211	490	29	16	25	12

The incidence of the severe asthma population undergoing mAb therapy testing positive for COVID-19 was higher compared to the severe asthma population not undergoing mAb therapy with a corresponding OR of 2.95 (95% CI = 1.72 to 5.05, p < 0.01). However, the incidence of the severe asthma population who tested positive for COVID-19 undergoing mAb therapy did not affect progression to severe COVID-19 disease and admission compared to those not on a mAb. This is summarised in Table 3.

**Table 3:** Summary of outcome measures and statistical significance. COVID-19: coronavirus disease 2019; mAb: monoclonal antibody; OR: odds ratio; CI: confidence interval

	OR	CI
COVID-19-positive patients on mAb vs. non-mAb	2.95	1.72-5.05
COVID-19-positive patients on mAb vs. non-mAb progressing to severe COVID-19	0.22	0.66-0.82
COVID-19-positive patients on mAb vs. non-mAb requiring admission	0.27	0.09-0.83

In severe asthma patients on mAb therapy who tested positive for COVID-19, the average BMI was higher (35.9 vs. 33.3). In addition, more patients were current smokers (6.1% vs. 2.9%) compared to those who tested negative.

## Discussion

A key finding from this retrospective Liverpool study is the low incidence of COVID-19 (7.8%) in the severe asthma population. This links with other studies which document similar findings [11]. The low occurrence of COVID-19 seen in the Liverpool severe asthma population is consistent with an early study conducted in Wuhan which did not list asthma as a comorbidity but rather reported diabetes mellitus and hypertension as more common comorbidities [12,13]. Studies conducted in Italy and Belgium further support this notion [6,14]. This finding could be attributed to numerous causes. For example, it is thought an inflammatory type 2 response associated with cytokines (IL-4, IL-5, and IL-13) and the clinical inflammatory phenotype of asthma marked by eosinophilia may have protective effects through the activation of the antiviral host defence and promotion of viral clearance [15,16]. Moreover, the presence of eosinopenia was predominately seen in the peripheral blood among those testing positive for SARS-CoV-2 [12]. Perhaps, a pathological environment favouring a high eosinophil count may provide protection against COVID-19 such as the case evident in the airways of severe asthmatic patients.

In the Liverpool severe asthmatic population testing positive for SARS-CoV-2, patients were older, had a higher BMI, and a larger proportion of the patients were female compared to severe asthmatics testing negative for COVID-19. A similar finding was seen in previous studies which reported that patients with asthma and COVID-19 were older, predominately female, and obese [17,18]. The higher prevalence of women with COVID-19 has also been reported in previous research [19-21]. This could be due to a higher baseline prevalence of asthma in females in the Liverpool severe asthma population. The incidence of bronchiectasis in COVID-19-positive and negative patients was similar (8.5% vs. 8.7%). Current smokers in the COVID-19-positive cohort were lower (11.9%) compared to severe asthmatics testing negative (13.1%). However, the proportion of smokers in the severe COVID-19 group was higher compared to patients who did not progress to severe COVID-19 disease (14.3% vs. 11.1%). This implies smoking may increase the likelihood of disease progression and severity which is consistent with prior studies [22]. A lower FEV1 is observed in the severe asthma population testing positive for COVID-19 compared to patients testing negative (2.14 vs. 2.26). This suggests a lower FEV1 may be attributable to a higher risk of COVID-19 infection which was a key finding in a recent study [23].

A hallmark in COVID-19 pathogenesis involves a key receptor named angiotensin-converting enzyme-2 (ACE2). ACE2 is a cellular receptor utilised by the spike protein of SARS-CoV-2 to gain host entry [24]. This is facilitated by the membrane-bound protease serine 2 (TMPRSS2) [25,26]. Therefore, it follows comorbidities that increase the upregulation of the ACE2 and TMPRSS2 genes would thereby increase the susceptibility to COVID-19 infection. For instance, smoking promotes the upregulation of the ACE2 gene which has been linked with increased severity of illness [22]. This was shown in the Liverpool severe asthma population. For patients on mAb therapy, a higher proportion of patients testing positive for COVID-19 were current smokers and had a higher BMI. Educating patients on the importance of smoking cessation and weight loss is crucial in this subgroup.

A further critical finding of this Liverpool study is the incidence of COVID-19 in the severe asthma patient population is higher in patients taking a mAb (13.5%) compared to those not taking a mAb (5%). A similar finding was seen in another study which highlighted that severe asthma patients on biologics were at higher risk of COVID-19 compared to the general Dutch population [27]. The mechanism of action of biologics varies in relation to the type of treatment used. For instance, mepolizumab and reslizumab work to counteract eosinophil expansion through inhibition of IL-5 inducing eosinophil cytotoxicity [28]. Considering the role of eosinophils in adaptive immunity and antiviral activity it follows depletion in numbers may increase the susceptibility to infections. Consequently, it can be deduced that careful monitoring of the eosinophil count is required for severe asthmatics under mAb treatment as low levels may increase the risk of COVID-19 infection. In addition, it was shown that severe asthma patients taking mAbs were not at a higher risk of severe COVID-19 disease, hospitalisation, or ICU admission in the Liverpool severe asthma population. A comparable result was found in a multitude of studies indicating no relationship between biologic use in severe asthmatics and poorer clinical outcomes of COVID-19 [29,30]. The current evidence suggests it is safe to continue biological therapy in severe asthmatics with COVID-19 with no impact on disease severity.

A key strength of this study is the array of resources that were used to gather the data for this study which involved the use of a dashboard, ICE, eExchange and emails. This allowed for a more robust collection of primary and secondary care data. Objective measures of severity of disease (observation, intubation, CPAP) was another strength of this study. A potential limitation of this study is COVID-19 infection rates in the Liverpool severe asthma population may have been underestimated due to patients not adhering to regular testing, strict shielding, and missed positive cases in the community due to nonreporting. In the future, telephone confirmation will be more appropriate to confirm presumed negative COVID-19 patients.

## Conclusions

This study is among the first to review the incidence and severity of COVID-19 disease in the Liverpool severe asthma population undergoing biologic therapy. A fundamental finding is that severe asthmatic patients have a low incidence of COVID-19. Results also indicate patients on mAb treatment may be at a higher risk of COVID-19 infection. Furthermore, the presence of obesity or lifestyle factors such as smoking while on mAb therapy may increase the susceptibility to COVID-19 infection. It is also shown that severe asthmatics undergoing mAb treatment do not have a higher risk of severe disease progression or admission. This is consistent with previous studies. This research supports the current practice of continuing biologic therapy during COVID-19 infection in severe asthmatic patients.
